# Computational approach for assessing the involvement of SMYD2 protein in human cancers using TCGA data

**DOI:** 10.1186/s43141-023-00594-7

**Published:** 2023-11-16

**Authors:** Arvind Kumar Yadav, Tiratha Raj Singh

**Affiliations:** 1 Department of Biotechnology and Bioinformatics, Jaypee University of Information Technology, Solan-173234, Himachal Pradesh, India; 2 Centre of Excellence in Healthcare Technologies and Informatics (CHETI), Department of Biotechnology and Bioinformatics, Jaypee University of Information Technology, Solan-173234, Himachal Pradesh, India

**Keywords:** SMYD2, Cancer, mRNA expression, Survival analysis, Mutational analysis, Pathways analysis

## Abstract

**Background:**

SMYD2 is a protein of the SET and MYND domain-containing family SMYD. It can methylate the lysine residue of various histone and nonhistone cancer-related proteins and plays a critical role in tumorigenesis. Although emerging evidence supports the association of SMYD2 in the progression of cancers, but its definitive effect is not yet clear. Therefore, further study of the gene in relation with cancer progression needs to be conducted. In the current study, investigators used TCGA data to determine the potential carcinogenic effect of SMYD2 in 11 cancer types. The transcriptional expression, survival rate, mutations, enriched pathways, and Gene Ontology of the SMYD2 were explored using different bioinformatics tools and servers. In addition, we also examined the correlation between SMYD2 gene expression and immunocyte infiltration in multiple cancer types.

**Results:**

Findings revealed that higher expression of SMYD2 was significantly correlated with cancer incidents. In CESC and KIRC, the mRNA expression of SMYD2 was significantly correlated with overall survival (OS). In BRCA, KIRC, COAD, and HNSC, the mRNA expression of SMYD2 was significantly correlated with disease-free survival (DFS). We detected 15 missense, 4 truncating, 4 fusions, and 1 splice type of mutation. The expression of SMYD2 was significantly correlated with tumor purity and immunocyte infiltration in six cancer types. The gene GNPAT was highly associated with SMYD2. Significant pathways and Gene Ontology (GO) terms for co-expressed genes were associated to various processes linked with cancer formation.

**Conclusion:**

Collectively, our data-driven results may provide reasonably comprehensive insights for understanding the carcinogenic effect of SMYD2. It suggests that SMYD2 might be used as a significant target for identifying new biomarkers for various human tumors.

**Supplementary Information:**

The online version contains supplementary material available at 10.1186/s43141-023-00594-7.

## Background

Cancer has become a serious health burden which surpassed cardiovascular diseases as the second largest cause of death worldwide [1]. According to the latest press release by the WHO in 2020, there were 19.3 million new cancer patients diagnosed, and approximately, 10 million cancer deaths occurred globally [2]. The evolutions in genetic and epigenetic parameters promote tumorigenesis. Differentially expressed genes linked to cancer patient survival could be exploited as diagnostic markers for early cancer detection [3, 4]. Therefore, cancer investigation, identification of related biomarkers, and development of methods for active prevention are essential requirements for the early screening of cancer.

Protein methyltransferase (PMT) is a catalytic enzyme that helps in the transfer of the methyl group to its substrate with the help of methyl donor S-adenosyl-L-methionine (SAM). It plays a significant role in the regulation of epigenetic mechanisms and is involved in the methylation of various substrates [5, 6]. PMTs play a crucial role in transcriptional events through histone methylation and nonhistone methylation at the position of arginine or lysine residues. Protein lysine methyltransferases (PKMT) are a type of PMT that helps to transfer a methyl group to the lysine residue of the substrate protein. It has been described that overexpression of proteins from PKMTs was linked with different types of human cancers [7, 8].

SMYD2 is a protein from PKMTs implicated in tumorigenesis and can influence gene transcription through lysine methylation [9]. Numerous studies have revealed the activity of SMYD2 methylation to nonhistone proteins such as P53 and RB1 [10, 11]. The SMYD2-specific nonhistone substrates are significantly associated with the carcinogenicity [12, 13]. Numerous tumor-causing proteins, for example, P53 [14], heat shock protein (HSP90) [15], retinoblastoma (Rb) [16, 17], ERα [18], PTEN [19], PARP1 [20], and STAT3 [21] being methylate by SMYD2. Therefore, it has been evidenced that SMYD2 is an onco-related protein that can affect the function of cancer suppressor proteins. The data analysis demonstrated that higher expression of SMYD2 is present in a variety of human cancers, like breast, bladder, colorectal, cervical, esophageal, lymphoma, ovarian, head and neck, and pancreatic cancer [14, 16, 22].

In the present analysis, we systematically explored the SMYD2 expression and its clinical outcomes to evaluate its potential marker for cancer treatment. Various expression and patient survival datasets available on several online platforms were used for this analysis. We measured multiple factors, such as the difference in gene expression, survival value, gene mutations, phosphorylation, methylation, immune infiltration, and functional enrichment analysis to explore the potential molecular mechanisms of the oncogenic role of SMYD2 on pathogenesis. Collectivelly, we identified that SMYD2 was not only potential biomarker but also may be of promising therapeutic target for multiple cancers.

## Methods

### Gene expression analysis

Expression analysis of SMYD2 was performed by using two web servers TIMER2 (http://timer.cistrome.org/) [23] and GEPIA2 [24] (http://gepia2.cancer-pku.cn/#analysis). Both utilized the tumor and non-tumor expression data from The Cancer Genome Atlas (TCGA). Genotype-tissue expression (GTEx) data was used to perform the expression difference between the tumor tissues and normal tissues using statistical method analysis of variance (ANOVA) with the help of GEPIA2. Parameters for the assessment method were under the setting of log2FC of 1, *p*-value of 0.01, and “Match TCGA normal and GTEx data.” Moreover, the violin plots for the SMYD2 expression level in diverse stages of pathology (stages I–V) of all TCGA tumors were also obtained via the GEPIA2 [24]. The log2 [TPM (Transcripts per million) +1] transformed data were applied for the construction of violin plots.

### Survival prognosis analysis

Kaplan–Meier (K-M) plots were calculated to perform the survival analysis of cancer patients in TCGA cancers with the help of the GEPIA2 server [24]. The median score was used as a cutoff to divide the high-expression and low-expression cohorts. Then, samples with expression level higher than 50% were considered high-expression cohorts, and lower than 50% were considered low-expression cohort. With the help of K-M plots, we analyzed the overall survival (OS) and disease-free survival (DFS) for 11 cancer types. The log-rank test also called Mentel-Cox test was utilized for hypothesis test and *p*-value < 0.05 was considered statistically significant for all survival analyses. Additionally, the hazard ratio (HR) with 95% confidence intervals was also computed.

### Promoter methylation analysis

The promoter methylation analysis in multiple cancer patients has been studied in TCGA dataset by using the UALCAN (http://ualcan.path.uab.edu/analysis-prot.html) [25]. It provides the facilities to estimate the cancer-related multi-omics data and assists to analyze the expression of proteins present in the Clinical Proteomic Tumor Analysis Consortium (CPTAC) as well as TGCA data. To generate the analysis results, default parameters were used. The statistical method Wilcoxon rank-sum test was used for the methylation differential analysis.

### Genetic alteration analysis

The cBioPortal (https://www.cbioportal.org/) has a large-scale web resource for cancer research [26, 27]. This study is based on TCGA data, so genetic alteration was performed by selecting the “TCGA Pan-Cancer Atlas Studies.” All TCGA tumors were examined for the frequency of alteration, type of mutations, and DNA copy number alterations by using 4617 samples. We also analyzed the differences between TCGA cancer patients with and without SMYD2 mutations in terms of overall, disease-free, disease-specific, and progression-free survival. To create the K-M graphs, the log rank of the *p*-value was utilized with the significant level of <0.05.

### Immune cell infiltration analysis

By choosing the immune cells such as cancer-associated fibroblasts cell and CD8 + T cells, the TIMER2 web server [23] was performed the association analysis between immune cell infiltration and SMYD2 expression across all tumor types. Immune infiltration was estimated using a variety of methods such as CIBERSORT-ABS, TIMER, CIBERSORT, EPIC, QUANTISEQ, MCPCOUNTER, and XCELL. The partial correlation and *p*-values were calculated by using the correlation test Spearman with the purity-adjustment parameter. The *p*-value < 0.05 was considered as statistically significant. The correlation output data was represented with the help of a scatter plot and heat maps.

### SMYD2-related gene enrichment analysis

Using the targeted data of normal and tumor tissues, the GEPIA2 server was utilized for finding the top 100 targeted genes associated with SMYD2. In this study, Enrichr web (https://maayanlab.cloud/Enrichr/) [28] was used for pathways and Gene Ontology (GO) analysis. Reactome 2022 and the Kyoto Encyclopedia of Genes and Genomes (KEGG) 2021 databases were utilized to define the signaling pathways. The SMYD2-correlated genes were categorized into three processes such as biological processes, cellular components, and molecular functions using different GO terms. The Cox *p*-value < 0.05 was considered statistically significant. The *q*-value (adjusted *p*-value) was calculated using the Benjamini–Hochberg method. Top ten enriched terms for input genes were displayed on bar charts based on the − log10 (*p*-value).

## Results

### Analysis of gene expression data

**Fig. 1:**
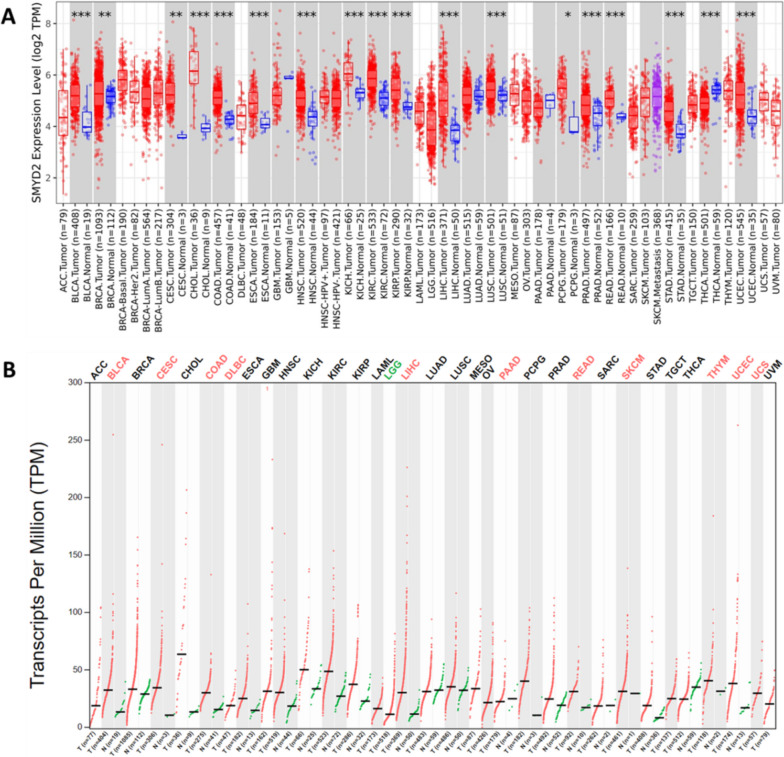
The difference in SMYD2 expression between the normal tissues and tumor tissues for all cancers of TCGA. **A** SMYD2 expression levels were analyzed for all cancers available in TCGA. The expression level of the tumor tissue is shown with red color box, and the normal tissue expression is shown as a blue color box. The stars represent the statistical significance level (*p < 0.05; **p < 0.01; ***p < 0.001). **B** Gene expression profile of SMYD2 was analyzed in tumor tissue (shown as red line) and normal tissue (shown as green line) samples using GEPIA2. The cancer names with significantly expressed are marked with red, lower expressed are marked with green, and non-expressed are marked with black color

To check the association of SMYD2 with cancers, the gene expression profile was analyzed in various normal and cancer types of tissues. The SMYD2 was overexpressed in most types of cancer (*p* < 0.001) as compared to the corresponding normal tissues (Fig. 1A). The *X*-axis shows the SMYD2 expression in log2 fold change values, whereas *Y*-axis shows the tissue types where SMYD2 is expressed. Analysis of TCGA datasets by using the GEPIA2 database also showed similar SMYD2 expression in bladder carcinoma, colon adenocarcinoma, diffuse large B-cell lymphoma, cervical squamous cell carcinoma, liver hepatocellular carcinoma, pancreatic adenocarcinoma, rectum adenocarcinoma, thymoma, uterine corpus endometrial carcinoma, skin cutaneous melanoma, and uterine carcinosarcoma (Fig. 1B). From here, we selected only the cancers reported with the overexpression of SMYD2 and associated with cancer progression. These cancer types are bladder, cervical, colon, breast, lymphoid, esophageal, liver, head and neck, kidney, ovarian, and pancreatic cancer.

**Fig. 2:**
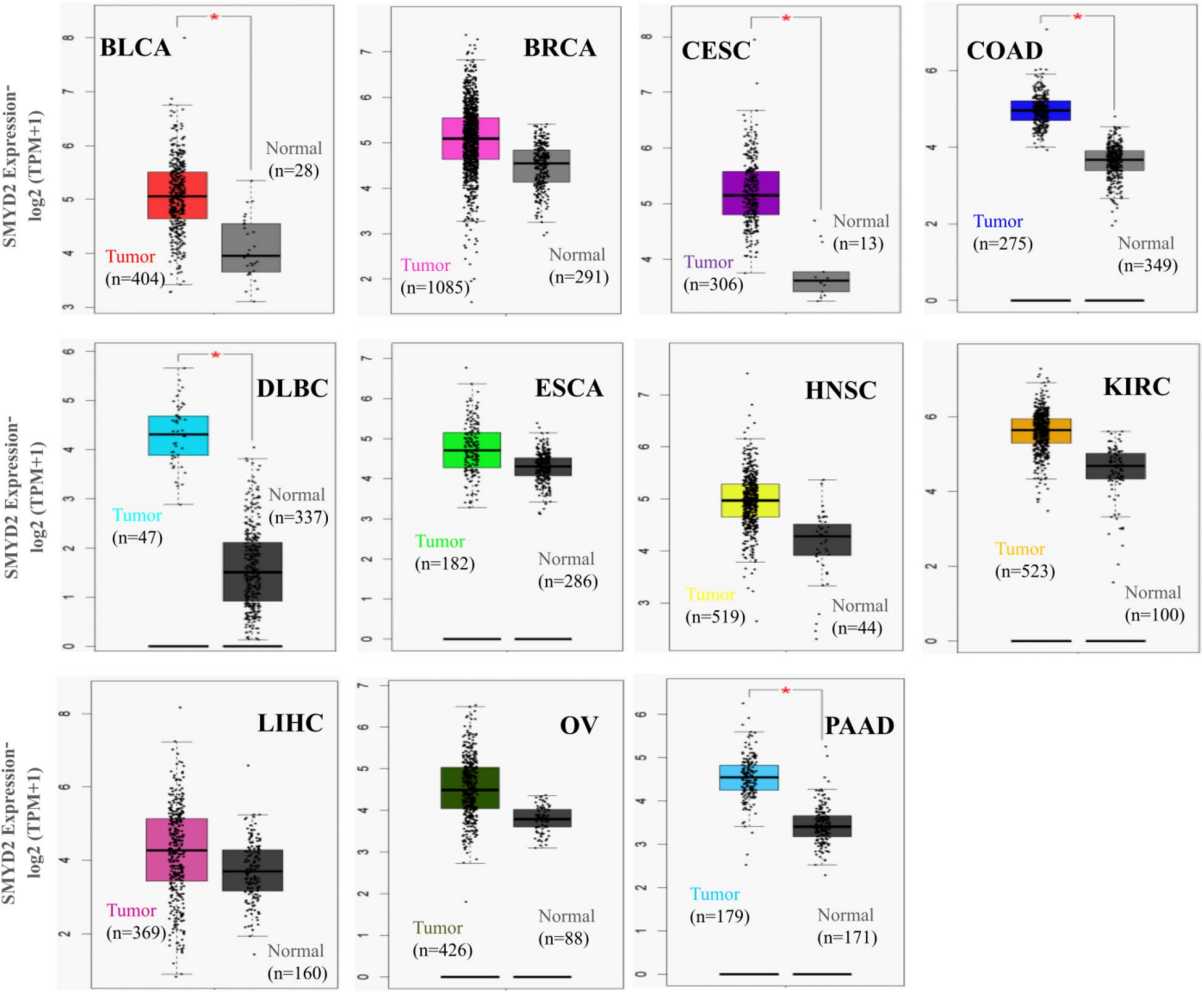
The box plot shows the SMYD2 expression in different cancers. The expression of SMYD2 in all cancer tissues was compared to the equivalent normal tissues using the GTEx as a control database. The expression of SMYD2 in the tumor tissues is shown in different colors, while corresponding normal tissues are shown in gray color. The line within the box represents the median, and the outliers are plotted as individual points. The statistically significant difference (*p* < 0.01) was marked with a red color asterisk (*)

We next used GEPIA2 to compare the expression difference of SMYD2 in the GTEx dataset as a control. Tumors and normal tissues of 11 cancer types, such as “breast invasive carcinoma (BRCA), bladder urothelial carcinoma (BLCA), colon adenocarcinoma (COAD), cervical squamous cell carcinoma, endocervical adenocarcinoma (CESC), lymphoid neoplasm diffuse large B-cell lymphoma (DLBC), head and neck squamous cell carcinoma (HNSC), esophageal carcinoma (ESCA), kidney renal clear cell carcinoma (KIRC),”liver hepatocellular carcinoma (LIHC), pancreatic adenocarcinoma (PAAD), and ovarian serous cystadenocarcinoma (OV), were considered for the evaluation of SMYD2 expression differences. The box plots were created to represent the expression of normal and tumor tissues. In comparison to normal tissues, all tumor types showed higher expression. The significant expression difference (*p*-value < 0.01) was observed in the BLCA, CESC, COAD, DLBC, and PAAD (Fig. 2).

**Fig. 3:**
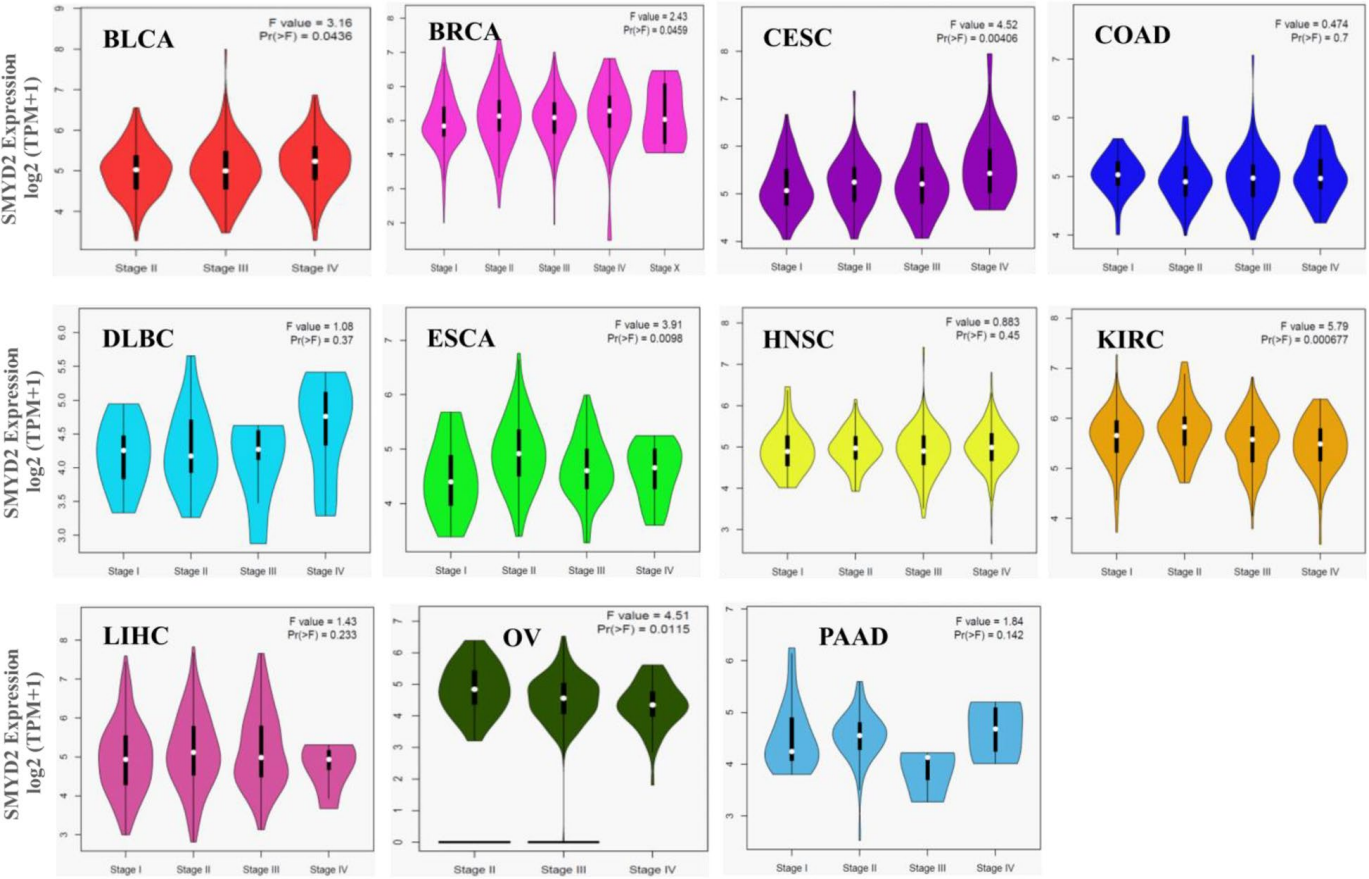
Violin plots representing the levels of SMYD2 expression between different pathological stages (stages I–V) based on the TCGA dataset by applying the log-scale as Log2 (TPM + 1). The height of the violin indicates the range of values observed for that stage. The central line inside each violin represents the median value for that stage. White dots denote median classification accuracies. The violin's shape represents the probability density function of the data distribution

Furthermore, we used to study the correlation between the cancer pathological stages (stages I–V) and SMYD2 expression. The violin plots for the pathological stages of all 11 cancer types are shown in Fig. 3. The width of each violin corresponds to the density of data points at that particular stage, with wider violins indicating a higher data density. The expression of SMYD2 varied more than six orders of magnitude in maximum cancer types. The height of each violin indicates the range of values observed for that stage. This analysis suggested that SMYD2 has a promoting role in the occurrence and progression of cancer.

### Analysis of survival data

**Fig. 4:**
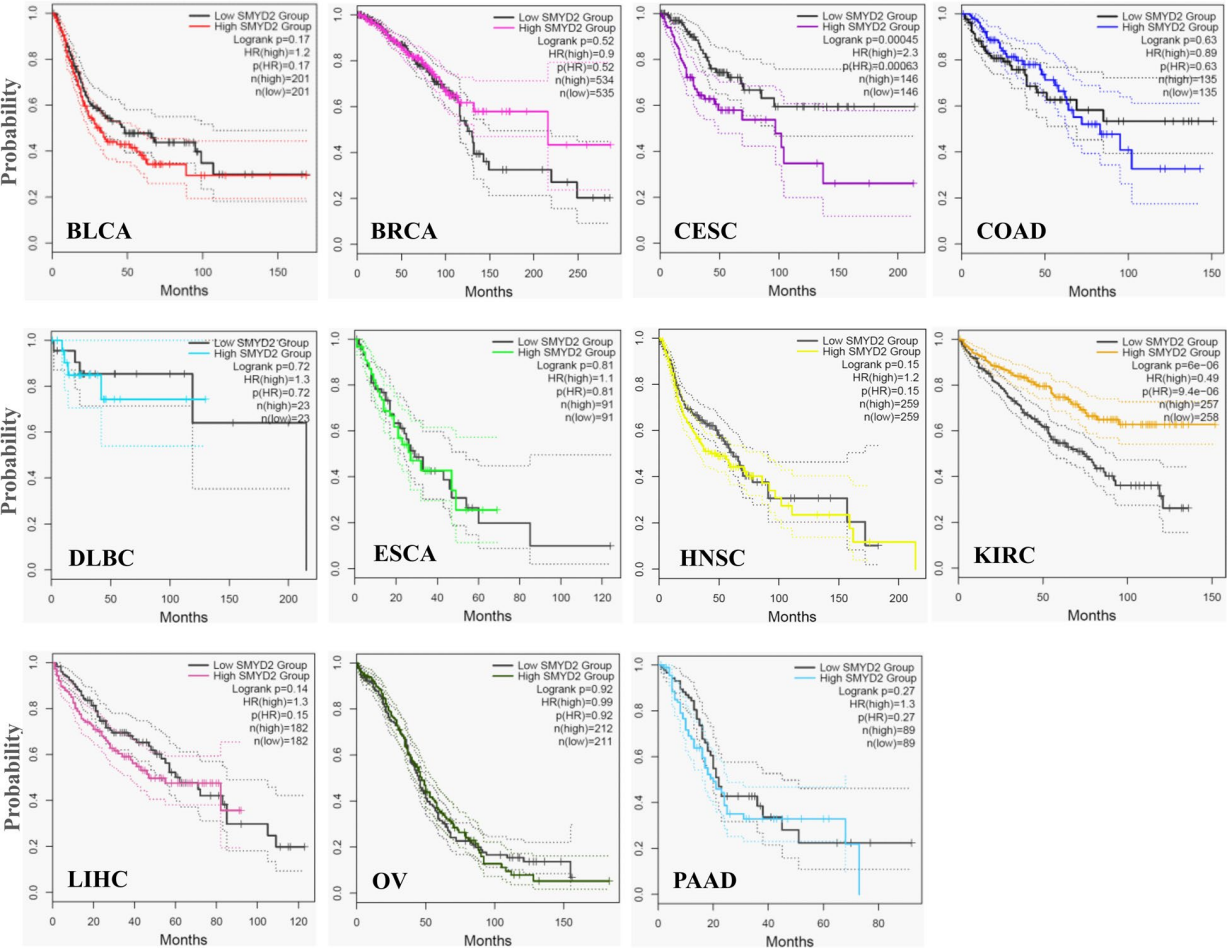
K-M plots show the relationship between the high expression group (color line) and low expression group (black line) of SMYD2 gene expression with the overall survival of patients in multiple cancers. The dotted lines show the minimum and maximum values of the survival average

**Fig. 5:**
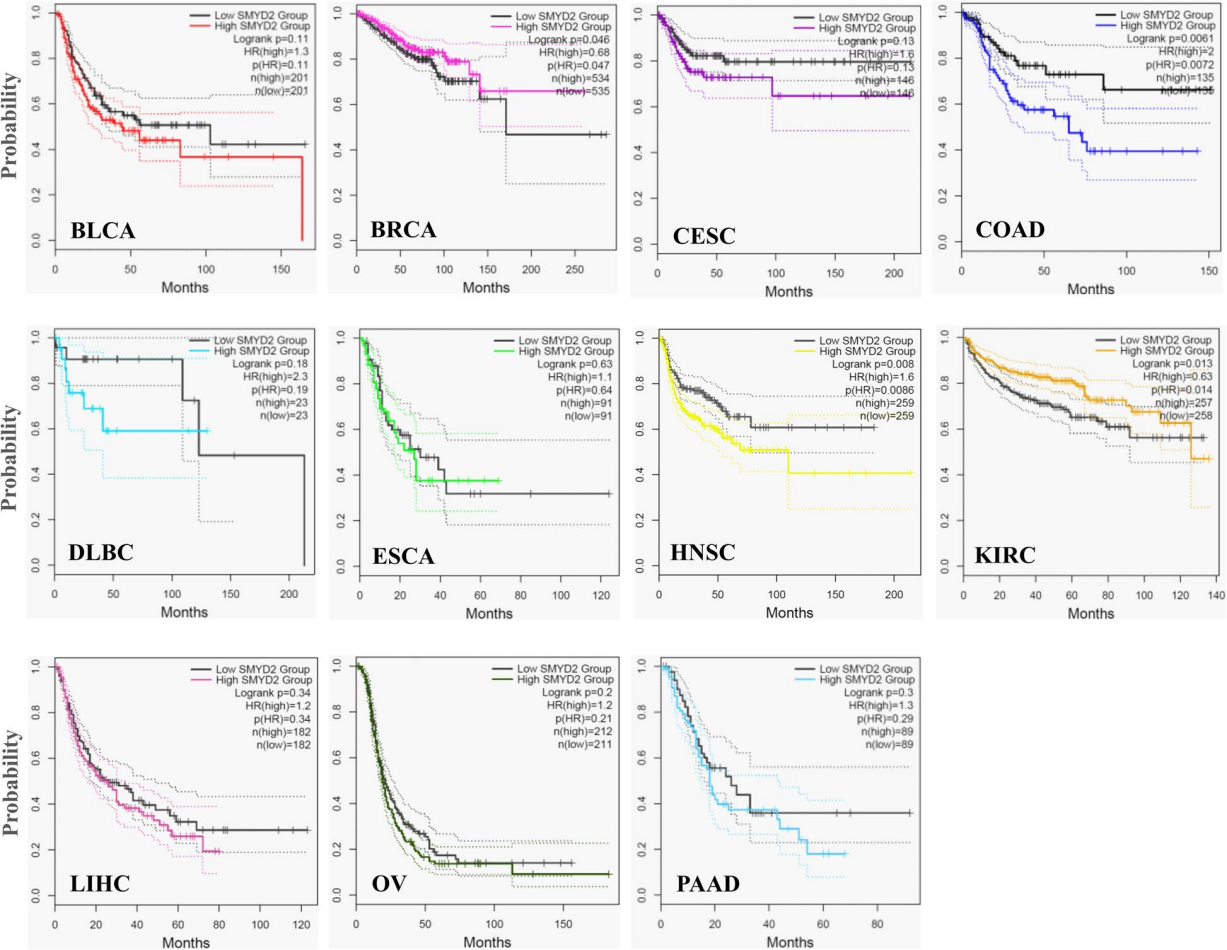
K-M plots show the relationship between high (color) and low (black) SMYD2 gene expression with disease-free survival of patients with multiple cancers. The dotted lines show the minimum and maximum values of the survival average

To explore the critical efficiency of SMYD2 in the survival of various cancer cases, we used GEPIA2 to evaluate survival data and establish an association between cancer patient survival and RNA expression SMYD2. The K-M plots for overall survival and disease-free survival analysis for all types of cancer were analyzed. A high level of SMYD2 in CESC (HR 2.3, *p* = 0.00045) and a lower level of SMYD2 in KIRC (HR 0.49, *p* = 6E-06) were significantly correlated with the OS of cancer patients (Fig. 4). The lower level of SMYD2 in BRCA (HR 0.68, *p* = 0.046) and KIRC (HR 0.63, *p* = 0.013) and a higher level of SMYD2 in COAD (HR 2, *p* = 0.0061) and HNSC (HR 1.6,* p* = 0.008) were significantly correlated with DFS (Fig. 5). The significantly correlated high level of SMYD2 expression indicated that patients with higher SMYD2 expression tend to live longer than those with lower SMYD2 expression, vice versa. The low SMYD2 expression group had a greater survival rate as compared to the higher expression group in maximum cancer types for both the OS and DFS.

### Analysis of DNA methylation data

**Fig. 6:**
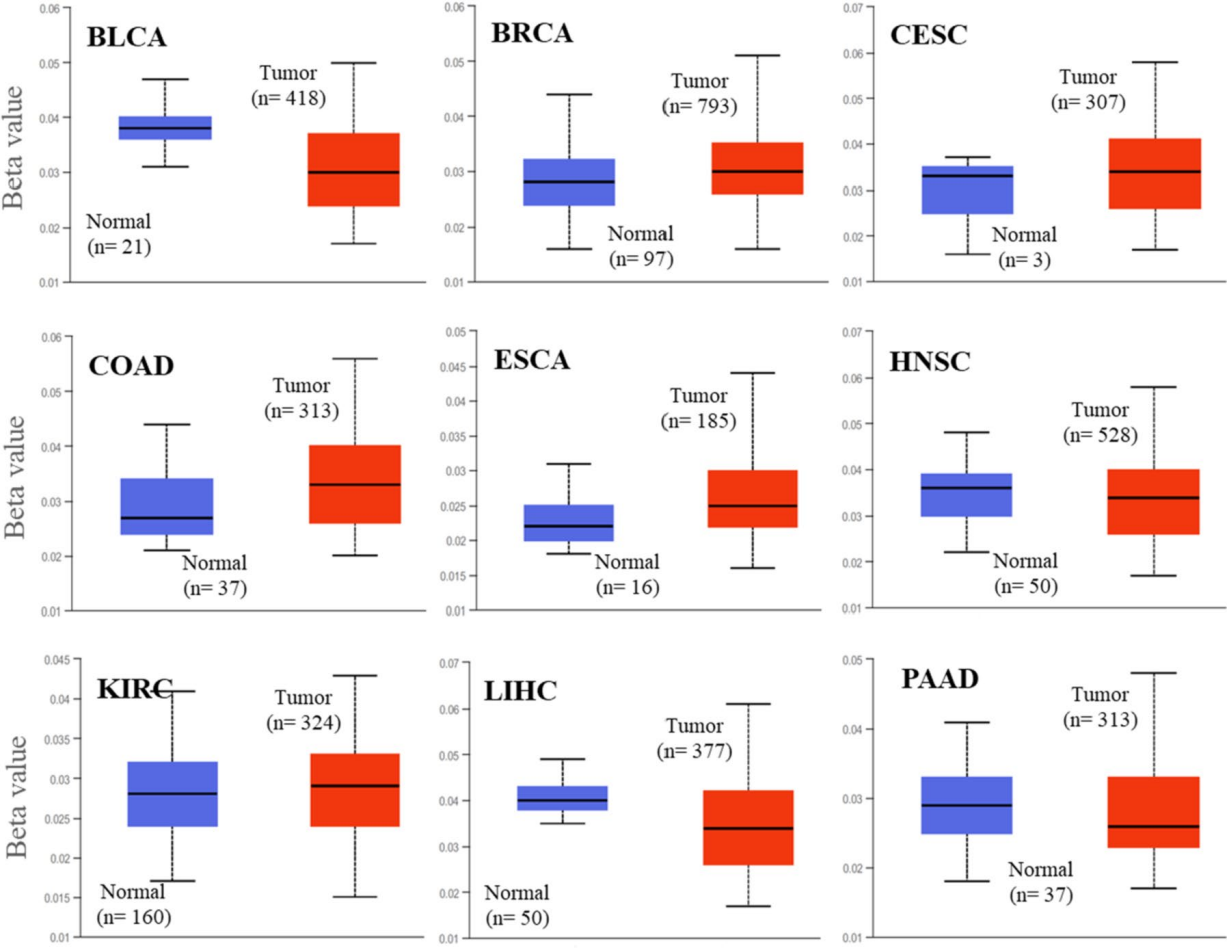
Promoter methylation level of SMYD2 gene in normal (blue) and tumor (red) tissues of various cancers. The level of promoter methylation is expressed as a box plot. The *y*-axis shows the DNA methylation level in terms of beta value

DNA methylation is an important epigenetic regulator of gene expression [29, 30]. Multiple malignancies have been found to have distinct and abnormal hypermethylation of CpG-rich regions (called CpG islands) or whole-genome hypermethylation [31, 32]. Hence, we discovered a possible correlation between SMYD2 expression and methylation in a range of cancers. The level of DNA methylation is represented in terms of beta values, which range from 0 (non-methylated) to 1 (fully methylated). The beta value towards one signifies the higher methylation level. In BRCA, CESC, COAD, ESCA, and KIRC cancers, the promoter methylation level was found higher. The reduced methylation level was observed in BLCA, HNSC, LIHC, and PAAD tumors compared to their counterparts of normal tissues in the UALCAN analysis using the TCGA dataset (Fig. 6).

### Analysis of genetic alteration data

**Fig. 7:**
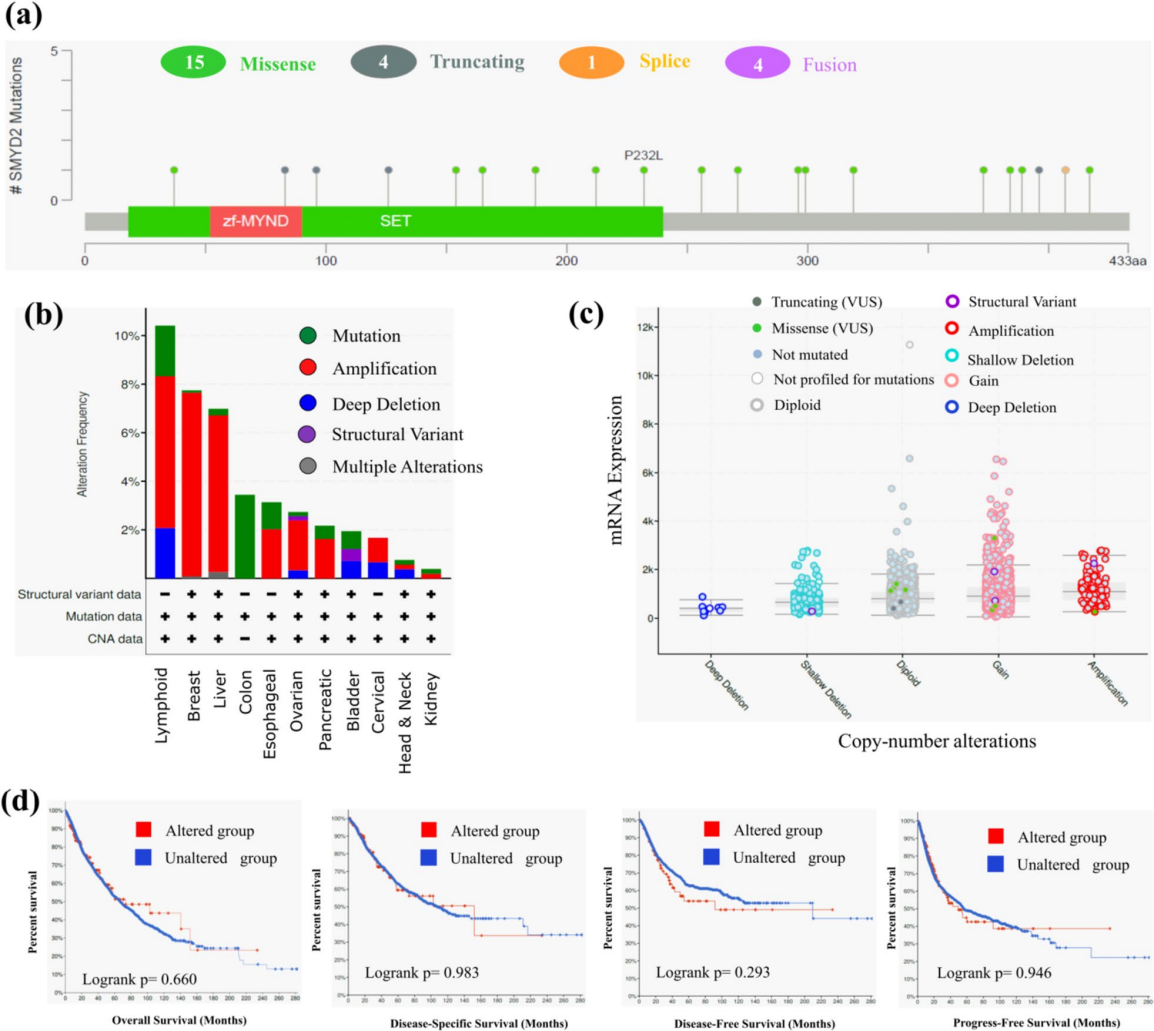
Genetic alteration and mutation of SMYD2. **a** The lollipop diagram depicts the alteration types within the SMYD2 protein sequence (1–433 AA). **b** Plot depicts genome alteration and alteration frequencies in the SMYD2 gene. **c** Correlation between copy number alteration of SMYD2 and mRNA expression present in TCGA data. **d** Association between mutation status and various conditions of survival analysis

The genetic alteration analysis of SMYD2 in several cancers was performed using cBioPortal. The SMYD2 gene mutations were searched in 4617 cancer samples from 11 different pan-cancer studies, including breast, cervical, bladder, colon, esophageal, kidney, head and neck, liver, lymphoid, pancreatic, and ovarian cancer. In SMYD2 433 amino acid long sequence, a total of 24 different mutations were detected, in which missense mutations (15 in number) are the most common form of genetic alteration (Fig. 7A). Maximum alteration frequency of SMYD2 (> 10%) seems in lymphoid cancer patients. The amplification type of copy number alterations was the main type in the lymphoid, breast, and liver cancer cases (Fig. 7B) (Supplementary Table 1). The deletion type of mutation was detected in several cancer patients. Additionally, as seen in the TCGA dataset, amplification and gain were more common (Fig. 7C). Moreover, the possible link between genetic variants of SMYD2 and survival prognosis across all TCGA cancer was also investigated. The survival analysis in comparison to SMYD2 with and without alteration was analyzed in disease-specific, overall, progression-free, and disease-free survival (Fig. 7D). The maximum survival difference was observed in disease-free survival analysis.

### Analysis of immune-infiltration data

Tumor-infiltrating immune cells are a chief constituent of the tumor microenvironment, and they play a crucial role in cancer progression, invasion, and metastasis [33, 34]. Cancer-related fibroblasts correlated to cancer in the stroma of the tumor microenvironment have been discovered to play a role in the functional regulation of immune cells infiltrating malignancies [35–37]. Here, we used various algorithms such as CIBERSORT, TIMER, XCELL, MCPCOUNTER, CIBERSORT-ABS, EPIC, and QUANTISEQ to study the correlation between immune cell infiltration and SMYD2 expression in multiple cancers types.

**Fig. 8:**
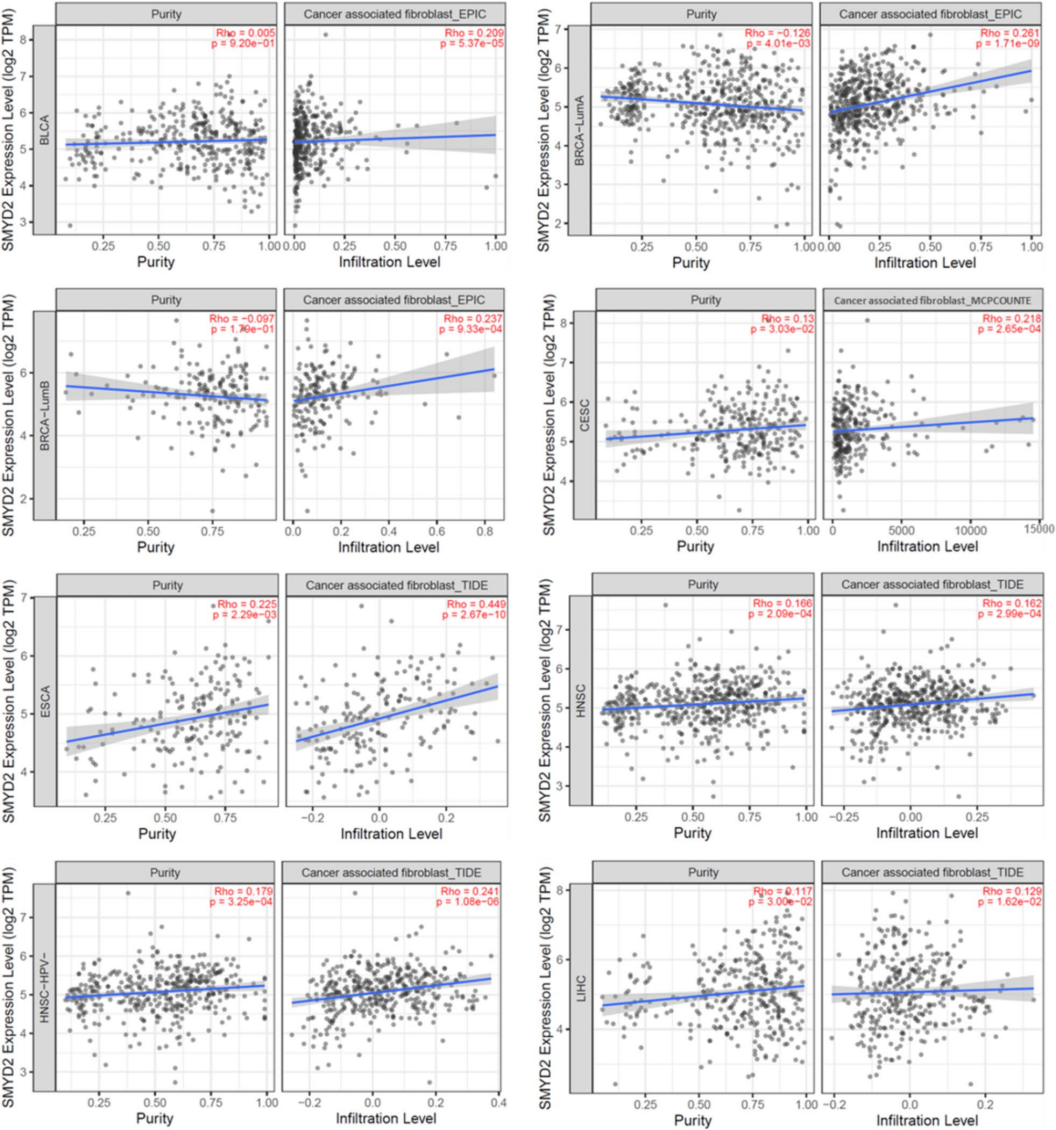
Investigation of correlation between immune cell infiltration of cancer-associated fibroblasts and SMYD2 expression in multiple cancer types

Analysis revealed that statistically significant positive correlation (*p* < 0.05 and Rho > 0) was detected between CD8 + T-cell and SMYD2 expression for BLCA (Rho = 0.208, *p* = 5.37E-05), BRCA (Rho = 0.115, *p* = 0.0002), DLBC (Rho = 0.340, *p* = 0.031), KIRC (Rho = 0.262, *p* = 1.12E-08), and LIHC (Rho = 0.129, *p* = 0.016). The statistically significant negative correlation (*p* < 0.05 and Rho < 0) was observed between the expression of *SMYD2* and immune infiltration of CD8^+^ T cell in BRCA basal (Rho =  − 0.160, *p* = 0.034), CESC (Rho =  − 0.137, *p* = 0.023), COAD (Rho =  − 0.171, *p* = 0.004), ESCA (Rho =  − 0.287, *p* = 9.50E-05), HNSC (Rho =  − 0.220, *p* = 7.69E-07), and PAAD (Rho =  − 0.195, *p* = 0.010) cancers of TCGA based on most or at least one algorithm (Supplementary Table 2). Furthermore, for BLCA (Rho = 0.208, *p* = 5.37E-05), BRCA-LumA (Rho = 0.208, *p* = 5.37E-05), BRCA-LumB (Rho = 0.237, *p* = 0.0009), CESC (Rho = 0.217, *p* = 0.0002), ESCA (Rho = 0.449, *p* = 2.67E-10), HNSC (Rho = 0.162, *p* = 0.0002), HNSC-HPV − (Rho = 0.240, *p* = 1.08E-06), and LIHC, a statistically significant positive correlation was found between the infiltration value of cancer-related fibroblasts and *SMYD2* expression. The scatter plot for these tumors was created by using one algorithm as shown in Fig. 8. A statistically negative association was detected for DLBC (Rho =  − 0.336, *p* = 0.031), KIRC (Rho =  − 0.135, *p* = 0.004), and PAAD Rho =  − 0.200, *p* = 0.008) of TCGA tumors based on most or almost all algorithms (Supplementary Table 2).

### Enrichment analysis of SMYD2-related genes

To explore the SMYD2-related genes, GEPIA2 server was utilized to identify the first 100 correlated genes with SMYD2 by combining all TCGA tumor expression data (Supplementary Table 3). The highest correlation was found in glyceronephosphate O-acyltransferase (GNPAT) (*r* = 0.42), insulin-induced gene 2 (INSIG2) (*r* = 0.41), and Egl-9 family hypoxia-inducible factor 1 (EGLN1) (*r* = 0.40). Finally, we used the list of associated genes with SMYD2 in various cancers for an ontology-level analysis to identify the putative signaling pathways.

**Fig. 9:**
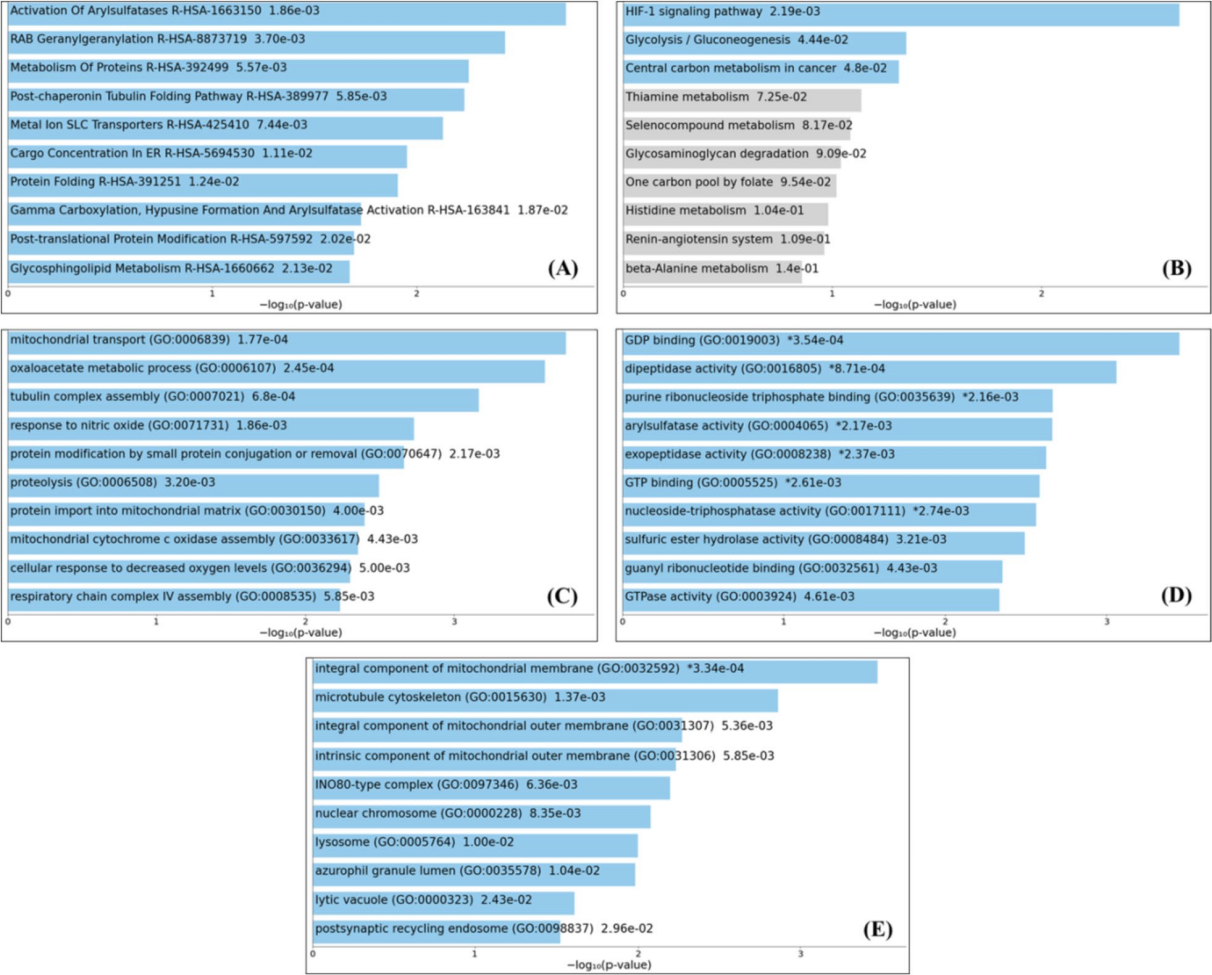
The bar charts show the top ten *SMYD2*-associated enriched terms. The best 100 *SMYD2*-associated genes obtained from GEPIA2 in TCGA projects were used for ontology analysis. The top ten enriched terms are displayed based on –log10 (p-value), with the actual p-value shown next to each term involved in the pathways and gene ontology (GO) functions. **A** RECTOME pathways 2022, **B** KEGG pathways 2021, **C** enrichment analysis of GO biological process 2021, **D** enrichment of GO molecular function 2021, and **E** enrichment of GO cellular component 2021. The more significant term is present on the top. Colored bars correspond to terms with significant *p*-values (< 0.05). An asterisk (*) next to the *p*-value represented the term which also has a significant adjusted *p*-value (< 0.05)

The first ten pathways from REACTOME and their interrelated genes were associated with the activation of arylsulfatases, RAB geranylgeranylation, metabolism of protein, post-chaperonin tubulin folding pathway, metal ion SLC transporters, cargo concentration in the ER, protein folding, gamma carboxylation and hypusin formation, post-translation protein modification, and glycosphingolipid metabolism. All these pathways showed a significant correlation with SMYD2 (Fig. 9A). The top ten KEGG pathways are mainly associated with a HIF-1 signaling pathway, glycolysis/gluconeogenesis, central carbon metabolism in cancer, thiamine metabolism, selenocompund metabolism, glycosaminoglycan degradation, one carbon pool by folate, histidine metabolism, renin-angiotensin system, and beta-alanine metabolism (Fig. 9B). A significant association was observed in the HIF-1 signaling pathway. Additionally, we also analyzed the GO terms for genes associated with SMYD2 to see their functions in biological processes, molecular functions, and cellular components. The recommended GO features mainly were involved in mitochondrial transport and oxaloacetate metabolic process in the biological processes category (Fig. 9C), guanosinediphosphate in the molecular function category (Fig. 9D), and an integral component of the mitochondrial membrane in the process of a cellular component category (Fig. 9E). All significant terms involved in pathways and GO functions along with *p*-value and *q*-value (adjusted *p*-value) are shown in Supplementary Table 4.

## Discussion

Cancer ranked as the second most prominent cause of global death that accounts for approximately 10 million deaths in 2020 worldwide [2]. The main cause for a higher number of cancer deaths is poor prognosis and advanced disease [38, 39]. So, the identification of effective biomarkers would be beneficial for early-stage diagnosis of cancer patients and will also help improve the treatment efficacy. Additionally, the proposed biological regulatory entities will help in the prognosis of multiple human malignancies. SMYD2 is a protein that takes part in the epigenetic modifications of the tumor suppressor gene and affects tumor transcription regulation by promoting nonhistone protein methylation [40]. Thus, it is important to explore how SMYD2 is associated with malignancies through common molecular mechanisms. Liu et al. studied the correlation of proteins from the SMYD family with cancer patients [40]. When we performed a literature review, we could not identify any integrated multicenter SMYD2 cancer analysis. Therefore, in the present study, we executed a multi-omics analysis to better understand the role of SMYD2 in diverse cancers.

In this systematic bioinformatics investigation of public datasets, we examined the expression value of SMYD2 across the tumors present in TCGA. The overexpression of SMYD2 was present in tumor tissues of all cancer types except a few compared to normal tissues. Furthermore, the overexpression of SMYD2 was observed in TCGA tumor tissues when GTEx data was used as a control. We found higher SMYD2 mRNA expression in all studied cancers. Overexpression of SMYD2 has been observed in several types of cancer, including breast cancer, lung cancer, and hepatocellular carcinoma [22]. The analysis of survival prognosis for the SMYD2 gene proposed discrete conclusions for tumors. Here, the GEPIA2 program was used to perform a statistical correlation between SMYD2 expressions and overall/disease-free survival rate of cancer patients. Results showed that a lower SMYD2 expression group has been significantly associated with a higher survival rate in the maximum type of cancer patients for both OS and DFS. The overexpression of the SMYD2 gene in tumor tissues was induced by a genetic mutation, CNAs, and epigenetic control. Moreover, mutational analysis from TCGA data suggested that CNA amplification was the most common type of alteration. The maximum alterations (> 10%) for SMYD2 were observed in lymphoid cancer. Further, correlated genes with SMYD2 were examined, and the top 100 genes were taken for pathways and GO analysis. In the REACTOME pathway analysis, the top pathways were associated with carcinogenesis [41–44]. For example, the activation of arylsulfatases is often decreased in cancer cells, leading to an accumulation of sulfated glycosaminoflycans (GAGs) in the extracellular matrix (ECM). This altered ECM composition can promote tumor growth and invasion by altering cell adhesion, migration, and signaling [45]. The abnormal RAB geranylgeranylation can be associated with cancer development and progression [46]. Furthermore, a study has shown that the post-chaperonin pathway for tubulin folding is associated with cancer [47]. The KEGG pathway analysis indicated a significant correlation with the HIF-1 signaling pathway involved in tumor progression and metastasis [48, 49]. The enriched GO terms involved in transport and metabolic process in the biological process category, guanosinediphosphate in molecular function category, and essential component of the mitochondrial membrane in the cellular component category were observed.

The present research shows the promising association of SMYD2 in multiple cancers through diverse publicly available bioinformatics tools and servers. Our integrated analysis shows that SMYD2 would be a potential biomarker for a wide range of cancers. The regulatory effect of SMYD2 on diverse cancers is different, though further experimental studies are desired to understand the complete molecular analysis of SMYD2 to identify its more effective biomarker role for cancer. Thus, SMYD2 can be used for the diagnosis of several cancers. Additionally, in vivo and in vitro research is necessary to clarify SMYD2 as a potential biomarker for cancer.

## Conclusion

In this comprehensive analysis, various bioinformatics databases and tools were used to elicit the SMYD2 expression, prognostics value, DNA methylation, mutation, CNAs, and correlated genes of SMYD2 in various human cancers. This comprehensive analysis shows that SMYD2 is significantly associated with multiple cancers. Heterogeneous data in TCGA were analyzed using extensive statistical and computational procedures that allowed us to reveal novel promising parameters for each examined cancer type. Additionally, our findings will give an enhanced understanding of the role of SMYD2 in the process of tumorigenesis and metastasis. The pan-cancer analysis provides a potential mechanism that suggested the expression of SMYD2 might modulate tumors. However, because these findings were based on data analysis, more experimental verification will be needed. In conclusion, SMYD2 would be a possible biomarker and a significant drug target for the prevention and management of human cancers.

### Supplementary Information

**Additional file 1: Supplementary Table 1.** Type of SMYD2 gene alterations present in different TCGA tumor samples with their alteration frequency aand alteration count.

**Additional file 2: Supplementary Table 2.** Correlation of SMYD2 expression with cancer associated fibroblast and immune cell infiltration in various cancer types using multiple algorithms.

**Additional file 3: Supplementary Table 3.** 100 correlated genes with SMYD2 by combining all TCGA tumor expression data.

**Additional file 4: Supplementary Table 4.** Significant pathways and functional enrichment analysis list with GO term of SMYD2 related genes.

## Data Availability

All data generated or analyzed during this study are included in this published article and its supplementary information files.

## Abbreviations

TCGA: The Cancer Genome Atlas
PMT: Protein methyltransferase
PKMT: Protein lysine methyltransferases
GTEx: Genotype-tissue expression
K-M: Kaplan-Meier
OS: Overall survival
DFS: Disease-free survival
BRCA: Breast invasive carcinoma
BLCA: Bladder urothelial carcinoma
COAD: Colon adenocarcinoma
CESC: Cervical squamous cell carcinoma and endocervical adenocarcinoma
DLBC: Lymphoid neoplasm diffuse large B-cell lymphoma
HNSC: Head and neck squamous cell carcinoma
ESCA: Esophageal carcinoma
KIRC: Kidney renal clear cell carcinoma
LIHC: Liver hepatocellular carcinoma
PAAD: Pancreatic adenocarcinoma
OV: Ovarian serous cystadenocarcinoma
