# Supplementary material for: Computational approach for assessing the involvement of SMYD2 protein in human cancers using TCGA data
Source: J Genet Eng Biotechnol. 2023 Nov 16;21:122. doi: 10.1186/s43141-023-00594-7 (PMC10654300; doi:10.1186/s43141-023-00594-7)
Supplement: Supplementary file 4 — Additional file 4: Supplementary Table 4. Significant pathways and functional enrichment analysis list with GO term of SMYD2 related genes. [file 43141_2023_594_MOESM4_ESM.docx]

**Supplementary Table 4:** Significant pathways and functional enrichment analysis list with GO term of SMYD2 related genes.

| **Term** | **p-value** | **q-value** |
| --- | --- | --- |
| **RECTOME Pathways** | | |
| Activation Of Arylsulfatases R-HSA-1663150 | 0.001862428 | 0.28068574 |
| RAB Geranylgeranylation R-HSA-8873719 | 0.003704472 | 0.28068574 |
| Metabolism Of Proteins R-HSA-392499 | 0.005567543 | 0.28068574 |
| Post-chaperonin Tubulin Folding Pathway R-HSA-389977 | 0.00584762 | 0.28068574 |
| Metal Ion SLC Transporters R-HSA-425410 | 0.007438995 | 0.2856574 |
| Cargo Concentration In ER R-HSA-5694530 | 0.011134522 | 0.33980545 |
| Protein Folding R-HSA-391251 | 0.01238874 | 0.33980545 |
| Gamma Carboxylation, Hypusine Formation And Arylsulfatase Activation R-HSA-163841 | 0.018714055 | 0.378438151 |
| Post-translational Protein Modification R-HSA-597592 | 0.020222681 | 0.378438151 |
| Glycosphingolipid Metabolism R-HSA-1660662 | 0.02131096 | 0.378438151 |
| **KEGG Pathways** | | |
| HIF-1 signaling pathway | 0.002190906 | 0.111736198 |
| Glycolysis / Gluconeogenesis | 0.044353776 | 0.494592481 |
| Central carbon metabolism in cancer | 0.047984787 | 0.494592481 |
| **GO Biological** | | |
| Mitochondrial transport (GO:0006839) | 0.000177012 | 0.077815177 |
| Oxaloacetate metabolic process (GO:0006107) | 0.000245087 | 0.077815177 |
| Tubulin complex assembly (GO:0007021) | 0.000679555 | 0.143839177 |
| Response to nitric oxide (GO:0071731) | 0.001862428 | 0.25053564 |
| Protein modification by small protein conjugation or removal (GO:0070647) | 0.002165765 | 0.25053564 |
| Proteolysis (GO:0006508) | 0.003202217 | 0.25053564 |
| Protein import into mitochondrial matrix (GO:0030150) | 0.004004046 | 0.25053564 |
| mitochondrial cytochrome c oxidase assembly (GO:0033617) | 0.0044345 | 0.25053564 |
| Cellular response to decreased oxygen levels (GO:0036294) | 0.005004401 | 0.25053564 |
| Respiratory chain complex IV assembly (GO:0008535) | 0.00584762 | 0.25053564 |
| **GO Molecular** | | |
| GDP binding (GO:0019003) | 0.000354195 | 0.044628576 |
| Dipeptidase activity (GO:0016805) | 0.000870867 | 0.049261757 |
| Purine ribonucleoside triphosphate binding (GO:0035639) | 0.002164601 | 0.049261757 |
| Arylsulfatase activity (GO:0004065) | 0.002165765 | 0.049261757 |
| Exopeptidase activity (GO:0008238) | 0.002370205 | 0.049261757 |
| GTP binding (GO:0005525) | 0.002607179 | 0.049261757 |
| Nucleoside-triphosphatase activity (GO:0017111) | 0.002736764 | 0.049261757 |
| Sulfuric ester hydrolase activity (GO:0008484) | 0.003205288 | 0.050483291 |
| Guanylribonucleotide binding (GO:0032561) | 0.004428742 | 0.058030422 |
| GTPase activity (GO:0003924) | 0.004605589 | 0.058030422 |
| GO Cellular |  |  |
| Integral component of mitochondrial membrane (GO:0032592) | 0.000334323 | 0.040453096 |
| Microtubule cytoskeleton (GO:0015630) | 0.001371658 | 0.082985311 |
| Integral component of mitochondrial outer membrane (GO:0031307) | 0.005356497 | 0.153876846 |
| Intrinsic component of mitochondrial outer membrane (GO:0031306) | 0.00584762 | 0.153876846 |
| INO80-type complex (GO:0097346) | 0.006358547 | 0.153876846 |
| Nuclear chromosome (GO:0000228) | 0.008346007 | 0.157408952 |
| Lysosome (GO:0005764) | 0.010033373 | 0.157408952 |
| Azurophil granule lumen (GO:0035578) | 0.010407203 | 0.157408952 |
| Lytic vacuole (GO:0000323) | 0.024264693 | 0.264603316 |
| Postsynaptic recycling endosome (GO:0098837) | 0.029630963 | 0.264603316 |
